# Frequency of Cardiovascular Complications and Its Association with Prognosis of COVID-19 Patients

**DOI:** 10.1155/2021/7073348

**Published:** 2021-12-08

**Authors:** Hossein Mazaherpour, Masoumeh Soofian, Elham Farahani, Fatemeh Masfari Farahani, Ehsanollah Ghaznavi Rad, Sakine Mazaherpour, Yousef Ramazani, Fatemeh Ashrafian, Amitis Ramezani

**Affiliations:** ^1^Arak University of Medical Sciences, Arak, Iran; ^2^Treatment Management of Social Security Organization of Khuzestan Province, Mahshahr, Iran; ^3^Kermanshah University of Medical Sciences, Kermanshah, Iran; ^4^Clinical Research Department, Pasteur Institute of Iran, Tehran, Iran

## Abstract

Coronavirus disease 2019 (COVID-19) may lead to acute respiratory disease; cardiovascular, gastrointestinal, and coagulation complications; and even death. One of the major complications is cardiovascular disorders, including arrhythmias, myocarditis, pericarditis, and acute coronary artery disease. The aim of this study was to evaluate the frequency of cardiovascular complications and to determine its association with the prognosis of COVID-19 patients. In a prospective analytic study, 137 hospitalized COVID-19 patients were enrolled. During hospitalization, an electrocardiogram (ECG) was performed every other day, and laboratory tests such as cardiac troponin I (cTnI) and creatine kinase-MB (CK-MB) were done 0, 6, and 12 hours after admission. These tests were repeated for patients with chest pain or ECG changes. Patients were categorized into three groups (improved, complicated, and expired patients) and assessed for the rate and type of arrhythmias, cardiac complications, lab tests, and outcomes of treatments. There was no significant relationship among the three groups related to primary arrhythmia and arrhythmias during treatment. The most common arrhythmia during hospitalization and after treatment was ST-T fragment changes. There was a significant age difference between the three groups (*P* = 0.001). There was a significant difference among the three groups for some underlying diseases, including diabetes mellitus (*P* = 0.003) and hyperlipidemia (*P* = 0.004). In our study, different types of arrhythmias had no association with patients' outcomes but age over 60 years, diabetes mellitus, and hyperlipidemia played an important role in the prognosis of COVID-19 cases.

## 1. Introduction

Since late 2019, a new form of the coronavirus family has caused a contagious respiratory disease in China and quickly led to pandemics of acute respiratory disease in many countries [1]. This single-stranded RNA virus, which is a member of the beta-coronavirus family and is known as coronavirus 2019 (COVID-19), appears with a variety of symptoms such as fever, dry cough, fatigue, and myalgia. Also, COVID-19 can lead to acute respiratory disease and extrapulmonary complications, e.g., cardiovascular, gastrointestinal, renal, coagulation, and skin disorders and even death [2]. One of the main complications of COVID-19 is cardiovascular disorders, which are caused by the presence of ACE-2 receptors in the lungs, heart, arteries, and kidneys [3]. SARS-CoV-2 virus uses this protein as a receptor to enter the cell [4]. Approximately 7.5% of myocardial cells express ACE-2 that can mediate the virus entry into the heart muscle and result in cardiotoxicity [5].

Some risk factors for cardiovascular complications of COVID-19 consist of old age, smoking, and underlying diseases, e.g., diabetes, hypertension, and cardiopulmonary disorders [6]. Underlying diseases associated with cardiac complications of COVID-19 increase the risk of mortality [7]. The results of previous researchers have revealed that cardiovascular complications of COVID-19 increase the mortality rate and the complications are related to the severity of the disease [2, 7]. Hypotension, tachycardia, bradycardia, arrhythmia, and sudden cardiac death have been reported as complications of severe viral illnesses such as SARS and influenza [8, 9]. Acute myocardial injury, arrhythmias, cardiogenic shock, acute coronary syndrome, venous thromboembolism, and even sudden deaths are other cardiovascular complications of COVID-19 [10, 11]. Some drug interactions in the treatment of COVID-19 are also becoming a serious concern and may put patients at risk for arrhythmias, cardiomyopathy, and sudden cardiac death [10].

Acute heart damage comes with increased cardiac troponin I (cTnI), echocardiography (ECG) abnormalities, and myocardial dysfunction [12]. In a retrospective study in China, 19.7% of 416 participant patients with COVID-19 had evidence of myocardial injury, and the mortality rate of patients with myocardial injury was 51.2%, while this rate was 4.5% in patients without myocardial injury [13, 14]. Moreover, different arrhythmias may occur following COVID-19, which can be due to this disease or different treatment regimens and interactions of them. The rate of arrhythmia in patients admitted to the ICU is higher than that in patients admitted to the general wards; in a study, it was 44.4% and 6.9%, respectively [1]. Subsequent studies have shown that these arrhythmias can be in the form of atrial fibrillation, conduction block, ventricular tachycardia, and ventricular fibrillation [13, 14].

On the other hand, in severe infections, the systemic inflammatory response and release of cytokines can cause vascular inflammation, instability of intravascular plaques, and increased risk of rupture, as well as increased production of vascular thrombosis and myocardial infarction [15]. Recent studies have indicated that the rate of acute myocardial injury among patients with COVID-19 is between 7.2% and 17% [1, 3]. In Wang et al.'s study, the values of cTnI and creatine kinase-MB (CK-MB) were higher in ICU patients with COVID-19 rather than patients in the general ward, indicating higher myocardial injury caused by this virus [1].

To the best of our knowledge, few researchers have assessed risk factors and types of cardiac complications following COVID-19. In this study, we aimed to evaluate the frequency of cardiovascular complications and its association with the prognosis of COVID-19 patients.

## 2. Methods

### 2.1. Study Design and Participants

This prospective analytic study evaluated all eligible patients with COVID-19 of Ayatollah Khansari Hospital and Amir Al-Momenin Hospital in Arak city, Iran, between June and October 2020. In this study, cardiac complications of COVID-19 were extracted from patients' files, electronic medical records, history, and physical exams by an infectious disease specialist.

Inclusion criteria were ages above 18 years old, consent for entrance in the study, and positive reverse transcription positive polymerase chain reaction (RT-PCR) for SARS-CoV-2 or clinical manifestations plus positive chest CT findings for COVID-19. Exclusion criteria were dissatisfaction with continuing this study and incomplete patient records. Eligible patients were divided into three groups: improved, complicated, and expired patients. The first group was described that improved in terms of clinical and vital signs after COVID-19 treatment and did not develop any specific complication at discharge time. The complicated group includes patients who developed complications related to the disease itself or due to treatment approaches during hospitalization. COVID-19 confirmed patients who died due to SARS COV-2 complications were classified as an expired group.

In the present study, cardiovascular complications including cardiac arrhythmias, myocardial infarction (MI), heart failure (HF), pericarditis, myocarditis, and increasing aortic and pulmonary artery diameter were assessed. These cardiovascular complications were diagnosed based on clinical signs and symptoms, physical examination, past medical history (especially underlying diseases), ECG, and chest CT scan. These findings were evaluated by an infectious disease specialist.

### 2.2. Data Collection

PCR sampling was performed through a sterile swab by one expert health care provider. A nasopharynx and oropharynx sample was obtained for each eligible patient on admission, and test result was usually prepared 48 hours later. Also, cTnI and CK-MB were sent at 0, 6, and 12 hours after admission.

ECG was performed on admission for all patients and repeated 6 and 12 hours later, followed by ECG daily or every other day according to physician's order. If the patient had chest pain or arrhythmia during hospitalization, the measurements of cTnI and CK-MB and also ECG were repeated. Then, ECGs and the result of cardiac enzyme tests were evaluated by a cardiologist. The cardiologist was unaware of the results of the treatment. Then, ECG findings and arrhythmias were classified into 9 categories: bradyarrhythmia, tachyarrhythmia, dysrhythmia, ST-T segment changes, QT changes, hemiblocks, bundle branch block (BBB), voltage changes, and R progression. Bradyarrhythmias included sinus bradycardia and AV blocks (grades I and II and complete block). Tachyarrhythmias included sinus tachycardia, atrial fibrillation (atrial fibrillation, atrial flutter, AVNRT, AVRT, and AT), and ventricular arrhythmias (VT, ventricular flutter). Dysrhythmias include PAC, PVC, and ST-T segment changes (e.g., ST depression and ST elevation, T tall, and T inversion). Hemiblocks included left anterior hemiblock (LAHB) and left posterior fascicular block (LPFB). BBBs included RBBB and LBBB. ECG voltage changes include LVH, RVH, and low voltage, and also the last category is R progression, which includes poor R progression and reverse R progression. Some arrhythmias and cardiac complications may be due to type of treatment regimen, and therefore, the types of antiviral drugs and antibiotics are divided into separate categories in this study.

Moreover, spiral chest CT was performed for all patients and then observed by a radiologist for evidence of cardiac complications including cardiomegaly, pericardial effusion, increased pulmonary, and aortic artery diameter.

This research was approved by the Research Ethics Commission of Arak University of Medical Sciences (approval ID: IR-ARAKMU.REC.1399.009). Written informed consents were obtained from the patient or one of the first-degree family members if the patient was unconscious.

### 2.3. Statistical Analysis

Data were collected, coded, and categorized, and then, data analysis was performed using SPSS software version 23. *P* values less than 0.05 were considered statistically significant. Descriptive data were calculated as frequency, frequency percentage, and analytical statistics. A chi-square test was used to assess the relationship between nominal variables. Fisher's exact test was used to evaluate the relationship between the risk factors and the complications of treatment. To analyze risk factors associated with cardiac complications of COVID-19 patients, multivariable logistic regression analyses with adjustment for important confounding variables, including age and sex, were performed. The multivariable logistic regression model was constructed using only variables with a significant *P* value (<0.05).

## 3. Results

In the present research, 4 patients were excluded due to incomplete medical records. Overall, 137 adult inpatients in Ayatollah Khansari Hospital and Amir Al-Momenin Hospital were enrolled in this study (improved group: *n* = 114 (83.1%), complicated: *n* = 18 (13.1%), and death: *n* = 5 (3.6%)). Most of the improved patients were 60 to 79 years old. This range was 40 to 59 years old for most patients of the complicated group, and 60 to 69 years of age for the expired patients. Therefore, there was a significant difference in age among the three groups (*P* = 0.001). There was no statistically significant difference in gender among the three groups (*P* = 0.54).

Blood pressure as a parameter of the vital sign had significant differences between each pair of the three groups (*P* = 0.005). But, there was no significant difference in body temperature, blood oxygen, and heart rate (Table 1).

There was a statistically significant difference among the three groups for some underlying diseases, including diabetes (*P* = 0.003) and hyperlipidemia (*P* = 0.004). Other underlying diseases such as hypertension, coronary artery disease, pulmonary disease, and cardiac procedures had no statistically significant difference. Information about underlying diseases of patients is summarized in Table 1.

There was not a statistically significant dissimilarity among the three groups for opioids, cigarette, and alcohol use (Table 1). Also, there was no significant relationship among the three groups related to primary arrhythmia and arrhythmias during treatment (Table 2). In the present study, the most common arrhythmia during hospitalization and after treatment was ST-T fragment changes. The findings of chest CT scans showed that there was no statistically significant difference for cardiomegaly (*P* = 0.348), increased diameter of the pulmonary artery (*P* = 0.474), increased diameter of the aorta (*P* = 0.903), and pericardial effusion (*P* = 0.295) among the three groups. The results of the current study showed no significant difference between patients for laboratory tests such as cTnI and CK-MB (*P* = 0.903 and *P* = 0.559, respectively).

Furthermore, there was a statistically significant difference among the three groups for the type of antibiotic treatment (*P* = 0.001), antivirals (*P* = 0.001), and corticosteroids (*P* = 0.003). The most common antibiotic therapy was levofloxacin, alone or in combination with vancomycin. The most common antiviral therapy was lopinavir/ritonavir (LPV/r), followed by interferon *β* 1a.

Finally, the treatment outcomes were as follows: 83.2% of patients were discharged without complications; 13.1% had complications, e.g., myocardial infarction, DVT/PTE, pulmonary fibrosis, unchanged blood oxygen level at discharge, pancreatitis, gastrointestinal bleeding (GIB), and pressure ulcer; and also, 3.6% of patients died during hospitalization.

There was no significant relationship between underlying diseases, mortality, and complications. Arrhythmias had no considerable relationship with the type of antiviral and antibiotic treatments. The relationship between arrhythmia during hospitalization and treatment with antibiotics and antiviral therapies was not statistically notable.

In the multivariable logistic regression analysis, we revealed that diabetes mellitus (OR, 1.8; 95% CI, 1.11-3.13; *P* = 0.02) and hyperlipidemia (OR, 1.69; 95% CI, 0.99-2.86; *P* = 0.04) were risk factors for the cardiovascular complications of COVID-19 patients.

## 4. Discussion

This study revealed the frequent risk factors and types of cardiovascular complications following COVID-19. As mentioned above, patients were statistically significantly different from each other based on their age group, and all expired patients were in the age range of 60-79 years. The result of Huang et al.'s study was accordant with our study and pointed out that age over 60 years is a risk factor for rapid progression and complications of the disease [2].

Blood pressure was significantly different among the three groups, while the differences in other parameters such as temperature, SpO_2_, and heart rate were not significant. Sixty percent (*n* = 3) of expired patients and 22.2% (*n* = 4) of complicated patients had hypertension, which is a known cause of poor prognosis among COVID-19 patients. Similarly, Zhou et al. assessed the risk factors for the increasing mortality rate and also COVID-19 progression. They found that hypertension is a risk factor for the poor prognosis and increased mortality among patients with COVID-19. In this study, the number of people with a history of hypertension as an underlying disease was higher than the number of people who presented with high blood pressure on admission. This seems to be due to the unstable vital signs and low blood pressure as well as the shock during hospitalization in some of the patients studied [6].

In the present study, there was a statistically significant difference among the three groups for underlying diseases including diabetes mellitus and hyperlipidemia. In regard to that, two studies reported that diabetes mellitus was a risk factor for poor prognosis and mortality in patients with COVID-19 [2, 6]. Moreover, Hariyanto and Kurniawan in a meta-analysis found that there is a significant relationship between dyslipidemia and progression of COVID-19 to a severe form, morbidity, and mortality, and also, the patients are prone to severe COVID-19 [16].

The results of this study showed a statistically significant difference among the three groups for the type of antibiotic therapy. The reason for this difference appears to be that WHO guidelines do not recommend antibiotic treatment for all mild to moderate COVID-19 unless a concomitant bacterial infection is suspected. It has been recommended that the physician begin antibiotic therapy in severe cases of COVID-19 [17]. In this study, 9 patients in the improved group did not receive any antibiotics and the rate of broad-spectrum antibiotics (levofloxacin plus vancomycin) was 34%, while all of the patients in the expired group (100%) received a broad-spectrum antibiotic.

The types of antiviral treatments were significantly different among the three groups. There is no consensus on the use of antiviral therapies for the treatment of COVID-19, and WHO guidelines do not confirm any of them [17]. Nevertheless, due to the uncertainty of the efficacy and outcomes of treatment with these antiviral drugs, in patients with severe COVID-19 and extensive pulmonary involvement, a combination therapy (such as remdesivir and antibiotic) was used. In addition, in the current study, the antiviral drugs used for the treatment of COVID-19 were constantly changed, and sometimes, some antiviral drugs were not available. Also, fewer numbers of complicated and expired patients may also be a possible reason for this considerable difference.

According to the findings of several studies, corticosteroid therapy is one of the suggested treatments for severe COVID-19 and acute respiratory distress syndrome (ARDS) [18]. In the current research, there was a statistically significant difference among the three groups for corticosteroid therapy. However, all expired patients received corticosteroid drugs during hospitalization; this rate was lower than both improved and complication groups. This is the possible cause of a statistically significant difference in corticosteroid intake among the three groups. There was no significant difference among the three groups regarding the types of cardiovascular complications following COVID-19. It was in accordance with the findings of our study. In the present study, arrhythmias, e.g., bradyarrhythmia, tachyarrhythmia, dysrhythmia, ST-T changes, BBB, voltage changes, hemiblock, and R progression, were found. ECG changes were not remarkably different among the three groups.

A meta-analysis by evaluating studies in several countries with COVID-19-related cardiac complications found that tachyarrhythmia from Asian countries was lower and the rate of bradyarrhythmias was higher than in other countries in the world [19]. Moreover, atrial fibrillation was the most common arrhythmia and 11.4% of patients developed bradyarrhythmia during treatment and this rate is higher than tachyarrhythmia during treatment (0.9%). In our study, the most common arrhythmia was ST-T segment changes. The reason for this difference seems to be related to the low number of patients in this study, the type of drugs used for treatment, and the presence of previous arrhythmias in the studied cases (10.5% of patients had ST-T changes on admission and before starting treatment).

## 5. Conclusion

In our study, various risk factors and underlying diseases such as age over 60 years, diabetes mellitus, and hyperlipidemia can play an important role in the prognosis, possible complications, and mortality of COVID-19 cases. In this study, different types of arrhythmias had no association with patients' outcomes.

## 6. Limitation

This study has some limitations including low sample size, the focus on just hospitalized patients, and without long-term follow-up of the effects of SARS-CoV-2 on the cardiovascular systems.

## Figures and Tables

**Table 1:** Patient characteristics and the frequency of underlying diseases.

Data	Group data	Improved *n* (%)	Complicated *n* (%)	Death *n* (%)	*P* value
Age groups	20-39	28 (24.6)	1 (5.6)	0 (0)	0.001
	40-59	32 (28.1)	14 (77.8)	0 (0)	
	60-79	39 (34.2)	3 (16.7)	5 (100)	
	>80	15 (13.2)	0 (0)	0 (0)	
Gender	Male	59 (51.8)	7 (38.9)	2 (40)	0.54
	Female	55 (48.2)	11 (61.1)	3 (60)	
Diabetes		26 (22.8)	11 (61.1)	2 (40)	0.003
Hypertension		39 (43.2)	9 (50)	3 (60)	0.245
Hyperlipidemia		25 (21.9)	10 (55.6)	0 (0)	0.004
Coronary arterial disease (CAD)		17 (14.9)	5 (27.8)	0 (0)	0.234
Pulmonary disease		13 (11.4)	1 (5.6)	1 (20)	0.612
Smoking		15 (13.2)	4 (22.2)	1 (20)	0.564
Opium		7 (6.1)	0 (0)	0 (0)	0.475
Alcohol		3 (2.6)	0 (0)	0 (0)	0.734
Fever		36 (31.6)	7 (38.9)	3 (60)	0.36
Blood pressure	Low	5 (4.4)	5 (27.8)	0 (0)	0.005
	Normal	75 (65.8)	9 (50)	2 (40)	
	High	34 (29.8)	4 (22.2)	3 (60)	
SpO_2_	>94%	19 (16.7)	1 (5.6)	0 (0)	0.35
	90-94%	36 (31.6)	4 (22.2)	1 (20)	
	<90%	59 (51.8)	13 (72.2)	4 (80)	
Pulse rate	Normal	56 (49.1)	10 (55.6)	1 (20)	0.36
	Tachycardia	58 (50.9)	8 (44.4)	4 (80)	

**Table 2:** Different types of arrhythmias among the three groups.

Time of incidence	Types of arrhythmias	Improved *n* (%)	Complicated *n* (%)	Death *n* (%)
Arrhythmias on admission	Without arrhythmias	82 (71.9)	9 (50)	2 (40)
	Bradyarrhythmias	3 (2.6)	2 (11.1)	0 (0)
	Tachyarrhythmias	8 (7)	1 (5.6)	1 (20)
	Dysrhythmias	2 (1.8)	0 (0)	0 (0)
	Changes ST-T	12 (10.5)	4 (22.2)	0 (0)
	BBB	1 (0.9)	1 (5.6)	0 (0)
	Voltage changes	1 (0.9)	1 (5.6)	1 (20)
	Hemiblock	1 (0.9)	0 (0)	0 (0)
	Changes Q-T	1 (0.9)	0 (0)	0 (0)
	R progression	3 (2.6)	0 (0)	1 (20)
	Without arrhythmias	41 (36)	5 (27.8)	0 (0)

Arrhythmias during hospitalization	Bradyarrhythmias	13 (11.4)	0 (0)	0 (0)
	Tachyarrhythmias	1 (0.9)	0 (0)	0 (0)
	Dysrhythmias	5 (4.4)	0 (0)	0 (0)
	Changes ST-T	19 (16.7)	4 (22.2)	1 (20)
	BBB	9 (7.9)	3 (16.7)	1 (20)
	Voltage changes	2 (1.8)	1 (5.6)	0 (0)
	Hemiblock	5 (4.4)	0 (0)	1 (20)
	Changes Q-T	14 (12.3)	2 (11.1)	1 (20)
	R progression	5 (4.4)	3 (16.7)	1 (20)

## Data Availability

The data that support the findings of this study are available from the corresponding author upon reasonable request.

## Abbreviations

COVID-19: Coronavirus disease 2019
ECG: Electrocardiogram
cTnI: Cardiac troponin I
CK-MB: Creatine kinase-MB
MI: Myocardial infarction
HF: Heart failure
BBB: Bundle branch block
LAHB: Left anterior hemiblock
LPFB: Left posterior fascicular block
LPV/r: Lopinavir/ritonavir
GIB: Gastrointestinal bleeding
ARDS: Acute respiratory distress syndrome.

## References

[1] Wang D., Hu B., Hu C. (2020). Clinical characteristics of 138 hospitalized patients with 2019 novel coronavirus-infected pneumonia in Wuhan, China. *JAMA*.

[2] Huang C., Wang Y., Li X. (2020). Clinical features of patients infected with 2019 novel coronavirus in Wuhan, China. *Lancet*.

[3] Turner A. J., Hiscox J. A., Hooper N. M. (2004). ACE2: from vasopeptidase to SARS virus receptor. *Trends in Pharmacological Sciences*.

[4] Hoffmann M., Kleine-Weber H., Schroeder S. (2020). SARS-CoV-2 cell entry depends on ACE2 and TMPRSS2 and is blocked by a clinically proven protease inhibitor. *Cell*.

[5] Zou X., Chen K., Zou J., Han P., Hao J., Han Z. (2020). Single-cell RNA-seq data analysis on the receptor ACE2 expression reveals the potential risk of different human organs vulnerable to 2019-nCoV infection. *Frontiers in Medicine*.

[6] Zhou F., Yu T., du R. (2020). Clinical course and risk factors for mortality of adult inpatients with COVID-19 in Wuhan, China: a retrospective cohort study. *Lancet*.

[7] China C. D. C. (2020). The epidemiological characteristics of an outbreak of 2019 novel coronavirus diseases (COVID-19) - China, 2020. *China CDC Weekly*.

[8] Li S. S., Cheng C., Fu C. (2003). Left ventricular performance in patients with severe acute respiratory syndrome: a 30-day echocardiographic follow-up study. *Circulation*.

[9] Harris J. E., Shah P. J., Korimilli V., Win H. (2019). Frequency of troponin elevations in patients with influenza infection during the 2017-2018 influenza season. *IJC Heart & Vasculature*.

[10] Dhakal B. P., Sweitzer N. K., Indik J. H., Acharya D., William P. (2020). SARS-CoV-2 infection and cardiovascular disease: COVID-19 heart. *Heart, Lung & Circulation*.

[11] Nishiga M., Wang D. W., Han Y., Lewis D. B., Wu J. C. (2020). COVID-19 and cardiovascular disease: from basic mechanisms to clinical perspectives. *Nature Reviews Cardiology*.

[12] Guo T., Fan Y., Chen M. (2020). Cardiovascular implications of fatal outcomes of patients with coronavirus disease 2019 (COVID-19). *JAMA Cardiology*.

[13] Shi S., Qin M., Shen B. (2020). Association of cardiac injury with mortality in hospitalized patients with COVID-19 in Wuhan, China. *JAMA Cardiology*.

[14] Zheng Y. Y., Ma Y. T., Zhang J. Y., Xie X. (2020). COVID-19 and the cardiovascular system. *Nature Reviews Cardiology*.

[15] Warren-Gash C., Hayward A. C., Hemingway H. (2012). Influenza Infection and risk of acute myocardial infarction in England and Wales: a CALIBER self-controlled case series study. *The Journal of Infectious Diseases*.

[16] Hariyanto T. I., Kurniawan A. (2020). Dyslipidemia is associated with severe coronavirus disease 2019 (COVID-19) infection. *Diabetes and Metabolic Syndrome: Clinical Research and Reviews*.

[17] World Health Organization (2020). Clinical management of COVID-19: interim guidance. https://apps.who.int/iris/bitstream/handle/10665/332196/WHO-2019-nCoV-clinical-2020.5-eng.pdf.

[18] COVID-19 treatment guidelines panel (2019). *Coronavirus disease 2019 (COVID-19) Treatment Guidelines*.

[19] Coromilas E. J., Kochav S., Goldenthal I. (2021). Worldwide survey of COVID-19 associated arrhythmias. *Circulation Arrhythmia and Electrophysiology*.

